# Correction: Heterogeneous organocatalysis: the proline case

**DOI:** 10.1039/d5ra90097b

**Published:** 2025-08-21

**Authors:** Gustavo Senra G. de Carvalho, Douglas C. Alcântara Pinto, Robson Corrêa da Silva, Fernando de Carvalho da Silva

**Affiliations:** a Universidade Federal Fluminense, Instituto de Química, Departamento de Química Orgânica, Campus do Valonguinho Niterói RJ Brazil senradcarvalho@gmail.com fcsilva@id.uff.br

## Abstract

Correction for ‘Heterogeneous organocatalysis: the proline case’ by Gustavo Senra G. de Carvalho *et al.*, *RSC Adv.*, 2025, **15**, 22469–22504, https://doi.org/10.1039/D5RA02331A.

The authors apologize that a related reference, presented here as ref. 1, was not cited in the original article. On page 22470, in the fifth paragraph of the Introduction, a citation to the reference should be added at the end of the sentence beginning “In fact, a very interesting molecule…”.

Ref. 16 in the original article should be ref. 16*a*, and the omitted reference should be ref. 16*b*:

“In fact, a very interesting molecule that well exemplifies the innovation brought by organocatalysis is exactly the amino acid proline, which has become a crucial component in examples of all catalytic strategies.^16*a*,*b*^”

The Royal Society of Chemistry apologises for these errors and any consequent inconvenience to authors and readers.
